# On Sight-Seeing

**Published:** 1895-09-28

**Authors:** 


### On Sight-Seeing.

Yes; we quite admit that it is bad form, and that to the unenthusiastic wanderer of the period the seeing of sights is one of the penalties of travel. We know that it is the growing custom to make holidays a matter of railways and hotels, to look on picture galleries and ancient castles as a bore, to accept mountains, glaciers, and natural phenomena as they stand without further investigation, and to look askance at the inquisitive traveller with his guide books and his continual questions. Yet this is a great pity, for of all the means for good in travel sight-seeing is one of the most efficacious.

The growing tendency shown by every class among brain workers to take a complete holiday every year is surely the outcome of some necessity of the times. Modern facilities for travel do not account for the rush to the sea, the moor, the Alps, which every August empties town. Rather we must take it that the intensity and excitement of work in some cases, and its monotony in others, drive people away in increasing numbers for rest and change.

Rest and change! How are they to be got? Pure air, bright sunshine, and outdoor exercise—these are things physical, and they can be attained; but it is mind-rest that is wanted, and a mental change that is required; and although in a long, long holiday all things, even forgetfulness of worries, come in time, this is not so in the shorter trips which alone fall to the lot of most professional and business men. For the mind works on at its old tasks even among new surroundings, and the very idleness of travel, which some people think is rest, does but give fresh opportunities for the busy brain to revolve old problems and to weary itself in disentangling jumbled skeins of thought, for which the busier life of cities left no time. This is not rest. In regard to its more trivial functions mere change of scene drives cerebral activity into fresh grooves, and breaks up those monotonous circles in which, under stress of business, it is wont to act; but man's mind cannot always by such simple measures be made to lie fallow; it cannot, by mere absence of its accustomed stimuli, be wiped clean like a slate and left unmarked by what has gone before. Scratched deeply upon it are the lines made by former thoughts, which will not come off, and can but be covered over and hidden away by new impressions. Even sleep is not always rest. Something fresh must be written on the mind or the old lines will reassert themselves; and here come in the great resources of sight seeing, adventure, and good companionship.

The beneficial effects of riding are not altogether due to its influence on the liver and the circulation; mountain climbing is not a mere matter of physical exercise; nor is scrambling over a glacier good only because of the stimulating effect of mountain air. It is by the effacement of normal lines of thought, the driving of the mind into fresh tracks, and also by the storing up for future reference and contemplation of new feelings and experiences, that these vivid moments are so useful.

"Exercise!" says the hypochondriac; "I am sick of it. I walk for miles and never lose my old sensations." We know well that men can both worry and think as actively while walking on the pavement or travelling by train as ever they do at the office desk. But it is another thing when the hounds are in full cry, or the grouse rise suddenly on the moor, or a slippery corner on the mountain is being passed. These are not moments when either sensations from within or worries from without can reach the consciousness; pains, miseries, and questions about markets vanish in thin air, and for the time the mind is wiped clean of its old thoughts. How automatic is the operation of walking on smooth pavements, and how small is the part taken by the mind in such a form of exercise is shown by the slightness of the impediment which suffices to cause a stumble. People, however, do not stumble over trifles in mountain climbing. Each footstep is a matter for observation and mental calculation. For hours together the mind may thus be strictly occupied in managing the feet, and the consciousness may thus be barred from those intruding thoughts which arise from the persistent action of the mind when unemployed. The glaciers and the mountains, however, are not for all; but all who travel can see the sights. The churches and museums, the old town-halls and curious bridges, the castles with their strange histories, the picture galleries and the collections of antiquities—in themselves, perhaps, dull things, and scorned by those who have already seen the world and found it empty—are full of usefulness to those who, if their holidays are to do them good, must not only find change of air and scene, but must displace the old thoughts they have within them, and put in new. Nor does the benefit of sight-seeing, however crude and tripper-like it be, end when the sight is seen. A holiday should do good not only at the time but afterwards, and not the least valuable of the things brought back from a holiday of sight-seeing is the crowd of memories which for months afterwards, in intervals between hours of work shall crop up and occupy the mind to the exclusion of *ennui* and dull care.

